# Integrating knowledge on biophysical and socioeconomic potential to map clusters for future milk production in Ethiopia

**DOI:** 10.1007/s11250-021-02695-2

**Published:** 2021-04-13

**Authors:** Oghaiki Asaah Ndambi, Tomaso Ceccarelli, Jelle Zijlstra, Michiel van Eupen, Tinsae Beyenne Berhanu, Adriaan Vernooij, Jan van der Lee

**Affiliations:** 1 Wageningen Livestock Research, Wageningen University and Research, PO Box 338, 6700 Wageningen, AH Netherlands; 2 Wageningen Environmental Research, Wageningen University and Research, Wageningen, Netherlands

**Keywords:** Sustainable dairy development, Dairy potential, Cluster ranking, Feed availability, Market quality

## Abstract

Despite growing milk demand and imports, market-oriented milk production and formal processing in Ethiopia is limited to areas around Addis Ababa, notwithstanding its competing land use demand. This study assessed biophysical and market potential for developing the dairy sector, characterizing Ethiopian dairy clusters. Biophysical data from geographic information system (GIS) sources and information from key informants were combined in mapping and ranking these clusters on milk production potential. Twenty-four indicators in six major categories were applied for this assessment: feed availability, environmental conditions for dairy cattle, current production status, access to inputs and services, output market access, and production expansion potential. Feed availability (fodder, crop residues, and agro-industrial by-products as well as land availability and affordability) were the main drivers for dairy development, followed by the current production status, mainly driven by number of (improved) dairy cattle and (formal) milk volumes. Dairy clusters close to Addis Ababa had the highest overall scores for development potential, mainly determined by local demand and access to inputs. For dairy sustainable dairy development in Ethiopia, companies seeking long-term opportunities may avoid the Addis Ababa area and develop dairy production and processing in other clusters especially in Amhara and Tigray regions, with good milk production potential but less developed market infrastructure. The combination of biophysical data and key informant knowledge offered key strengths in delivering valuable results within a short time span. It however requires a careful selection of knowledgeable key informants whose expertise cover a broad scope of the dairy value chain.

**Supplementary Information:**

The online version contains supplementary material available at 10.1007/s11250-021-02695-2.

## Background

Ethiopia has great opportunities for development of its dairy sector, as many parts of the country have a favorable agro-ecology for dairy cows. As second most populated country in Africa, Ethiopia is also one of the fastest-growing economies in the world (Yilma et al. 2011; Gray 2018). This growing population and increasing urbanization drive rising demand for dairy products by the growing middle class (Land O’Lakes 2010). However, milk consumption per capita in Ethiopia is amongst the lowest in the world, with considerable consequences on malnutrition, growth, and health, especially of young children. Increasing the diversity of diets is an important strategy to combat malnutrition, and milk plays an essential role herein (Lemma et al. 2017; D’Haene et al. 2019; Haileselassie et al. 2020). Such a malnutrition reduction strategy is hindered by the current infrastructure of the dairy value chain which is poorly developed, and over 95% of the milk is still traded informally. As a result, large volumes of milk are left uncollected from farmers, and the gap between supply and growing urban demand is widening. The resulting impeding value chain development and input market quality (Duncan et al. 2013) is evidenced by dairy production and processing capacity being constrained to the Greater Addis Ababa cluster, an area with many competing land use demands (Mekasha et al. 2014).

Investments in value chain infrastructure such as collection points and processing plants only take place in and around urban centers, which does not stimulate production and offtake in the agro-climatically more suitable rural areas. This leads to inefficient investments such as too many competing collection points near towns and underutilized processing plants (Bezie 2019). Furthermore, negative environmental consequences such as manure pollution and the need to transport feed over large distances from elsewhere in the country are encountered.

This study aims to map and characterize areas with the most suitable agro-ecological and socioeconomic potential to stimulate sustainable growth of the dairy sector in Ethiopia. Industry and development planners in Ethiopia need information about areas where milk could be produced most sustainably and to spread livelihood opportunities across farm households and chain actors in various regions (Getabalew et al. 2019; Tadesse and Yilma 2018). However, literature is scanty on methodology to identify and compare areas that do not only have the biophysical potential to efficiently produce significantly higher volumes of milk but also the market potential to match the demand for dairy products in a sustainable way. The study is important in providing options for addressing challenges related with dairying in the tropics, as described by Hernández-Castellano et al. (2019). Their study identified the poor adoption of temperate dairy farming systems in the tropics as a major challenge, especially as the weather conditions and infrastructure differ between the two regions. Key issues discussed by Hernández-Castellano et al. (2019) include reproduction and health challenges and also high greenhouse gas emissions per kg of milk produced. In this study, an attempt is made to address these challenges, by developing and applying a methodology to select, characterize, and rank dairy clusters in Ethiopia that match with current biophysical and socioeconomic conditions and also anticipate future changes in land use and the climate. In this way, the costs along the dairy chain and environmental footprints from dairy production could be reduced.

## Methodology

### Analytical framework

**Fig. 1:**
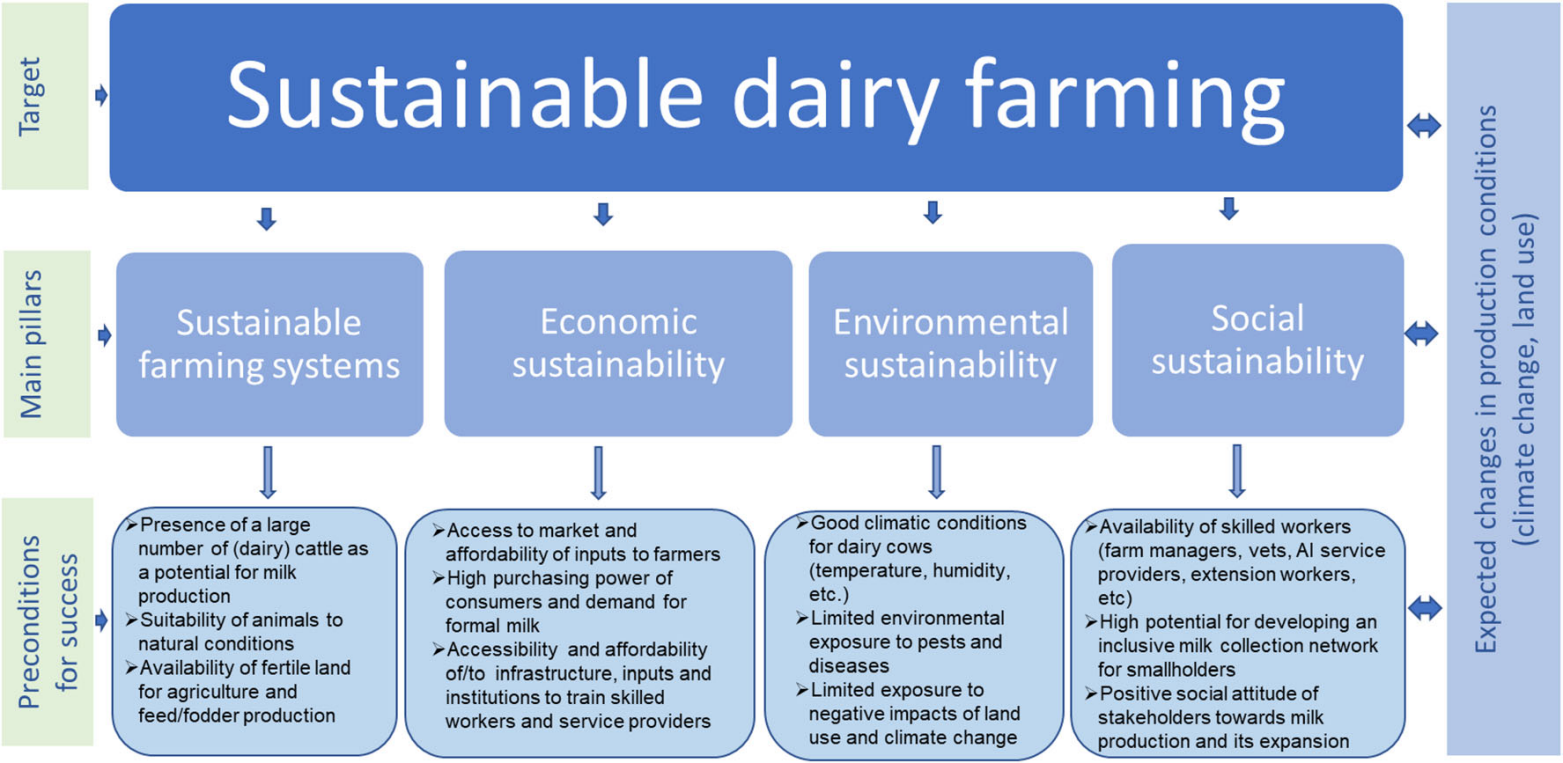
Analytical framework for assessing cluster potential for sustainable dairy farming

Dairy clusters are geographic concentrations of dairy farms and small and medium enterprises that facilitate the required linkages to input and output markets (van der Lee et al. 2018). The future development potential of clusters is depending on the prevailing farming system, market, and context conditions (including biophysical, institutional, and social conditions). Figure 1 portrays the analytical framework used in this study to identify cluster potential for sustainable dairy production. This study focuses on cattle, as cow milk constitutes the majority of milk produced in Ethiopia (Makoni et al. 2014). This framework has informed the selection of indicators that match the preconditions for sustainable milk production, divided into four sustainability pillars shown in Fig. 1. These four pillars have informed the selection of indicators for comparing the clusters as elaborated in Table 1. The four pillars of the sustainability framework are further elaborated in the next sections.

**Table 1:** Weighting of selected biophysical and socioeconomic indicators for cluster assessment

	Indicator	Sustainability pillar*	Score of 5 means:	Weighting factor	Explanation/background information for key informants
Biophysical indicators					
	a. Feed availability				
1	Availability and affordability of land	FS/Ec	Very positive	10	An indication for land sizes and ease of acquiring land for agriculture
2	Biomass production per ha (fodder potential)	FS	High	15	Biomass production ability and its change over time due changing climate
3	Availability of roughage and crop residues	FS	High	6	An indication for availability of grass, fodder crops (maize, sorghum, fodder beets, etc.), and crop residues for feed
4	Availability of by-product brewers waste	FS	High	2	Indicating potential to use by-products as a feed supplement
5	Availability of by-products for feed	FS	High	2	Indicating potential to use by-products such as oil seed cakes and wheat meal as concentrate feed
	b. Environmental conditions for cows				
6	Climate conditions for dairy cows	En	Ideal	5	Climate conditions based only on heat stress risk on an annual base (1 = little heat stress, 5 = more than 5 months with heat stress)
7	Animal health risks	En	Low	5	Based on the prevalence of ticks, FMD, and other diseases
	c. Current production status				
8	Milk volume (formal and informal)	FS	High	8	Total amount of milk produced in the cluster in kg
9	% of milk sold to formal market	Ec	High	3	% of milk delivered to milk processors. The rest is fed to calves, home consumed, or sold to neighbors, sometimes after local processing
10	Number of cattle	FS	High	2	Total number of cattle (including non-dairy)
11	Number of dairy cows	FS	High	5	Total number of dairy cows in the cluster
12	Number of improved dairy cows	FS	High	2	% of crossbreeds or exotic dairy cows in the cluster with a high milk yield potential
Socioeconomic indicators					
	d. Access to inputs and services				
13	Distance to closest feed factory	Ec.	Easy access	2	Indication for access to improved and likely cheaper feed due to reduced transportation costs
14	Skilled farm managers and farm workers	So	Easy access	1	Indication for ease of professionalization
15	Vet services	So	Easy access	2	Number of vet officers in the area and frequency of their visits to farmers
16	Insemination services	So	Easy access	1	Number of insemination workers in the area and their timeliness when called for insemination services
17	Private extension services	So	Easy access	2	Private services are mainly targeting commercial farmers
18	Electricity coverage	Ec	High	2	Is farm access to electricity reliable?
	e. Output market access				
19	Distance to main road	Ec	Short	5	Indication for ease of and cheaper transportation of milk and inputs
20	Distance to chilling center or processing plant	Ec	Short	5	Indication for effectiveness and ease of milk collection with a possibility to reduce transaction costs
	f. Production expansion potential in milk volume				
21	Expected growth in formal milk market	Ec	High	5	Examination of historical developments and possible future trends in formal milk demand, likely to affect production
22	Attitude of authorities towards increase in milk production	So	Very positive	2	Looking at the government’s long- and short-term plans for the area and how these are likely to increase or reduce milk production
23	Attitude of farmers to-wards production increase	So	Very positive	5	How common practices, traditions, and culture of farmers are likely to influence future milk production
24	Potential for future expansion of dairy farms	En	High	3	If farms have space and if the current land use and climate change trends show a future potential for milk production

*FS sustainable farming systems, *En* environmental sustainability, *Ec* economic sustainability, *So* social sustainability

#### An indicator framework of dairy sustainability

Indicators for sustainable agriculture have been classified into three pillars: economic, social, and environmental (Gan et al. 2017; Pretty 2008). The Sustainable Agriculture Initiative platform adds a fourth element: sustainable farming systems SAI Platform (2009) that focuses on the internal dynamics in the farming system.

##### Sustainable farming systems

These indicators consider the components and interactions within dairy farming systems, in terms of breeds, feeding, watering, and lodging of animals (Platform 2009; Vayssières et al. 2009). Meanwhile, Rademaker et al. (2017) emphasize that a farming system must be able to continue in time and that the farmer has a vital role to play in assuring this continuity. An available bank of well-performing animals is important for dairy development. Likewise, the sustainability of farming systems is enhanced by the availability of biomass, including roughages, cereals, and crop by-products that can be used in animal feeding (Vayssières et al. 2009). Cultivation methods for maintaining soil fertility such as erosion control and crop rotation are also important for sustainability (Nouwakpo et al. 2018).

##### Economic sustainability

These indicators look at possibilities to increase production while meeting the safety and quality requirements for products SAI Platform (2009). It also considers access to markets and possibilities of farmers to group themselves and acquire inputs or sell their milk (Yilma et al. 2011). Accessibility to and affordability of labor, land, and capital as well as infrastructure, such as roads and electricity, are essential to lower transaction costs in the dairy chain (van der Lee et al. 2018).

##### Environmental sustainability

These indicators consider soil fertility and water use as well as environmental impacts of dairy farming (waste management, pesticide, and fertilizer use) in the area SAI Platform (2009). For sustainable production, dairy farms must have access to water for drinking and feed production. Wherever rain fed systems are not reliable, irrigation is required. Irrigation could draw water from nearby water bodies (rivers, lakes, streams) or from groundwater. The ambient conditions for animals are important as high yielding dairy animals perform best under specific temperature, humidity, and altitude (Collier et al. 2006; Yilma et al. 2011; Bernabucci et al. 2014). The Temperature Humidity Index (THI), an index combining relative humidity and temperature, is used to relate heat stress effects to productivity of cows (Fodor et al. 2018). Besides the THI, environmental conditions might favor or disfavor the existence and proliferation of animal pests and diseases. One example is trypanosomiasis, a cattle disease (Steverding 2017) spread by tsetse flies, which reproduce in warm vegetative areas (Wamwiri and Changasi 2016).

##### Social sustainability

In this component, social and human capital are considered: the skills of workers and the incentives they are offered. Socially sustainable farms should provide a positive impact within their local community SAI Platform (2009). The government plays a key role in assuring the sustainability of the dairy sector by creating an attractive enabling environment which could stimulate job creation or increase access to farm inputs and product markets (Debele and Verschuur 2014; Yilma et al. 2011). The attitude of farmers and other stakeholders towards growth and market connectivity is considered important as it gives a general idea of the ease with which farmers would adopt initiatives to increase production (Gebreegziabher and Tadesse 2014).

#### Expected changes in production conditions

Current production conditions are subject to various changes that could affect future production potential. For example, Ethiopian woodlands are subject to serious environmental impacts due to heavy pressure causing them to shrink over time due to extraction of fuel and construction wood and expansion of cash crops like sesame in the northwest (Binyam et al. 2015). The two most anticipated changes by our key informants in Ethiopia are climate change and land use change, which hence have been elaborated below.

##### Climate change in Ethiopia

Climate change is likely to affect the future production potential of dairy clusters and thus needs to be considered in cluster scoring (Table 1). Location-specific scenarios have been used to assess climate change impacts on agriculture and livestock production systems in Ethiopia. Hadgu et al. (2015) indicated an expected increase in mean temperatures of 2 to 2.3 °C by 2030 and up to 2.7 °C in Northern Ethiopia by 2050. It is also predicted that climate change will lead to more intense rainfall in parts of Ethiopia, thus reducing the amount of land that can be used for agriculture and decreasing crop productivity because of waterlogging and flooding of farmland (DPPA 2007). This in turn means a reduction of livestock feed sourced through crop residues and by-products. Changes in rainfall and warmer temperatures may expand the geographical distribution and increase the survival of vectors such as flies and mosquitoes that are carriers of infectious livestock diseases (IFAD 2009; Thornton et al. 2009). Drought-associated losses of livestock have already been reported in the past 2 decades in the Borana Zone, southern Ethiopia (Ayal et al. 2018).

##### Land use changes in Ethiopia

Besides climate change, land use change could affect the dairy potential of a cluster. For example, shifting from grassland to cropland could reduce the availability of forage but could also increase the availability of crop residues and by-products for the dairy animal, the consequence being a replacement of better quality fodder by poorer quality fibrous residues. A study conducted in the northwestern Ethiopian highlands covered land use and land cover changes in Tigray (Kafta Humera area), Amhara (Metema area), and Benishangul-Gumuz (Sherkole area) over the 25-year period of 1985–2010 (Binyam et al. 2015). The main changes detected in all study areas were the conversion of dry (lowland) woodlands into agricultural land and, to a lesser extent, of shrubland and grazing land into agricultural land, or bare (fallow) land. This does not necessarily have direct negative implications on dairy production, as the prevailing livestock systems in this case rely on feed from crop residues and by-products. The reduction of grasslands and rangelands affects livestock production systems, especially where nomadic and semi-nomadic pastoralism prevails.

### Study procedure

**Fig. 2:**
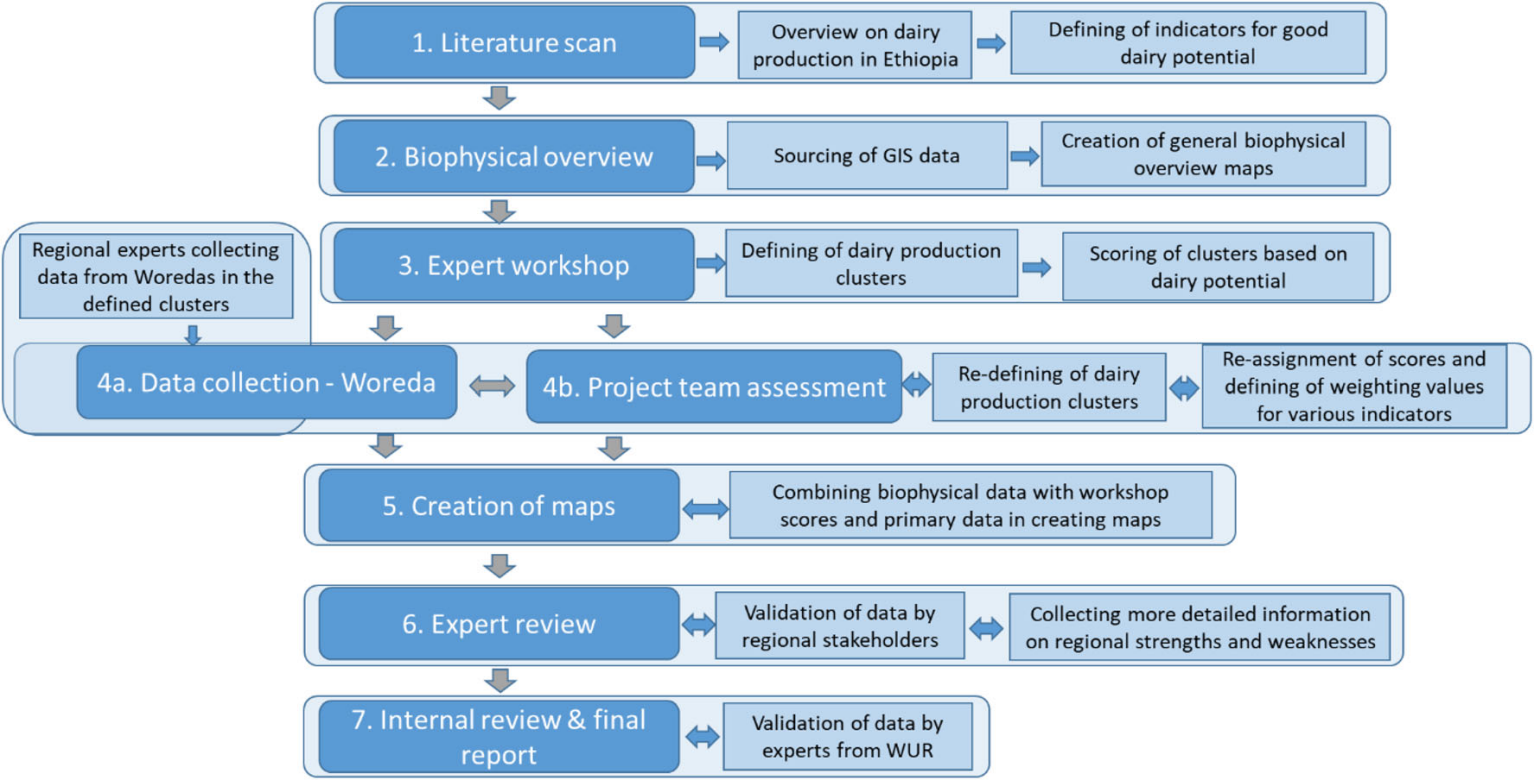
Steps in the applied approach

The study involved seven steps as shown in Fig. 2.

1. A *literature scan* was conducted from various secondary sources (reports, scientific articles, websites), giving an overview of dairy production and marketing chains and identifying a first draft list of indicators for distinguishing areas with a good dairy potential (later summarized in Table 1). Globally available open source geodata related to climate, soil, water, topography, land cover, agriculture, and anthropogenic factors were also evaluated.
2. *Biophysical potential maps* were created for the whole of Ethiopia. Based on the initial list of indicators, GIS maps were created. Three variables—total biomass, land cover/use, and heat stress index for dairy cows—were selected and mapped in combination as most important factors for delineating the dairy clusters (see Appendix 2 for further details). Additional supporting variables were also retained as possible means of verification of the dairy potential (e.g., population density, distance to cities, and especially cattle density, see Appendix 1).
3. In a *key informant workshop*, national and regional key informants reviewed the maps generated in step 2, to further delineate preliminary clusters with a high dairy production potential. Additional indicators, deemed important for the characterization of the clusters, were defined and scored by the key informants. Twenty-four dairy sustainability indicators were selected and clustered in six categories based on their relevance to dairy production: current production status, feed availability, expansion potential in milk volume, access to output markets, and access to inputs and services (Table 1). A total of sixteen key informants; twelve regional (three each from Tigray, Amhara, Oromia, and SNNP (Southern Nations, Nationalities and Peoples’) regions) and four (inter)national key informants scored these indicators with a range from 1 to 5 (five indicating the highest potential). The key informant workshop compensated for lack of secondary data on indicators to assess the potential of clusters. Key informants were briefed on the indicators using the explanations in the last column of Table 1, with an emphasis on land use and climate change impacts. They were also considered to be able to assess pros and cons on many criteria to come to a balanced evaluation of all the criteria. At the same time, we tried to avoid the negative aspect of using key informants: the risk of varying interpretations of the criteria. This was done by a harmonization instruction before the start of the assessment and by limited adjustments of results (called re-assignment in Fig. 2) by the four national and international key informants after the assessment.

This group of four key informants also assigned weighting factors to the indicators. After each key informant suggested a weight individually, differences in weight were discussed until a consensus was reached. The indicator scores were multiplied by the weighting factors, to calculate the weighted average score per category of indicators (Table 1).

4. Primary *data collection* on milk production, cow numbers, dairy infrastructure, and services by regional technicians from districts (woredas) within the clusters. Assessment of all primary data collected leads to a final delineation of the borders of clusters and to adjustments in the initial scores.
5. *Maps were created* using the new data collected from districts, showing the current milk production per square kilometer for the different clusters. The clusters were characterized using the biophysical potential data collected in step 2 and the primary data collected in step 4.
6. *Review meetings* involving regional and national key informants validated the results. These meetings provided additional information about strengths and weaknesses of the various clusters and performed a scoring on the potential of their region based on a list of 24 indicators described in Table 1. Based on this, the original scores from step 3 were adjusted.
7. A *report* (Ndambi et al. 2018) was written that underwent internal review by international experts and which formed the basis of this paper.

## Results

### Delineation of the clusters

**Fig. 3:**
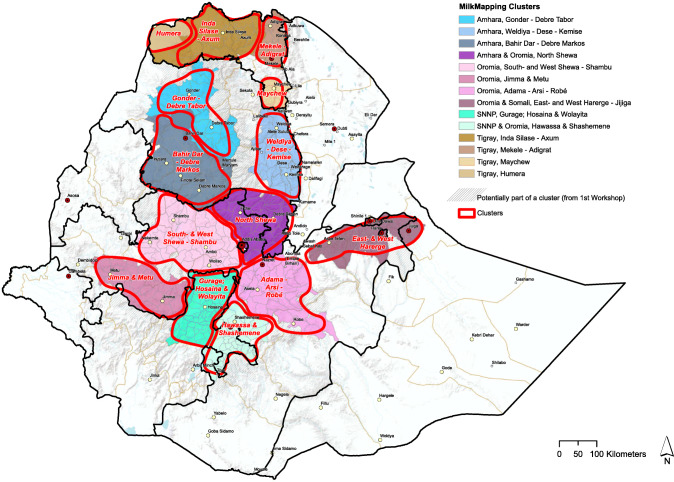
Milk production clusters showing district boarders

Fourteen dairy clusters with high potential to produce more milk in the future were identified. The clusters and their borders are shown in Fig. 3. Four clusters were fully located in Tigray, three in Amhara, three in Oromia, and one in SNNP, and another three clusters were interregional clusters crossing regional borders.

**Fig. 4:**
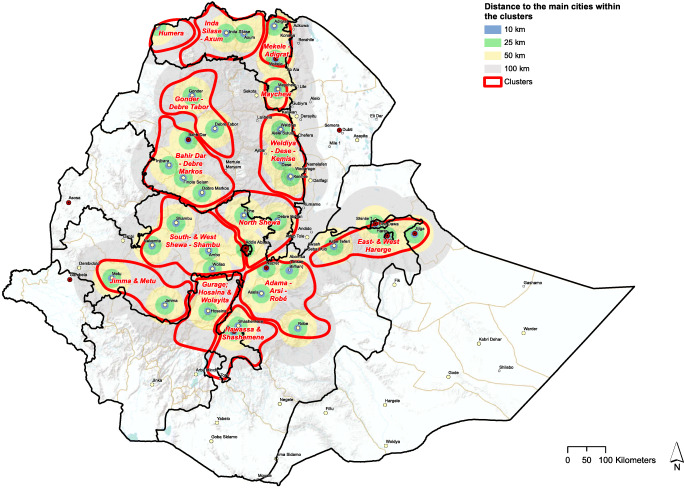
Distance to main cities and towns within the districts

The proximity to cities and major towns, indicating the proximity of markets for dairy products, was important in cluster mapping (Fig. 4).

### Biophysical potential

This section characterizes the clusters using maps produced from biophysical data. Only a selection of all indicators has been displayed, as due to the voluminous nature of illustrations, it was not possible to show illustrations for all individual indicators. However, by placing multiple layers in one map, all major indicators were displayed, while a number of minor indicators are discussed in “Socioeconomic potential.”

#### Milk volumes, cattle numbers, and tsetse density

#### Available land, land use, and biomass yield

Milk production requires land for feed production. This can be either grassland or cropland delivering fodder crops such as maize and alfalfa, or by-products from arable crops such as cereals, pulses, and oil seed crops. Table 2 and Fig. 7 show data about land use and land productivity to provide a better insight on the availability of fodder and feed ingredients.

**Table 2:** Biomass and land productivity per cluster

Region	Cluster name	Total land area per cluster (km^2^)	Total biomass productivity (t/day)	Relative biomass productivity (% of total in all clusters)	Average production of DM (t/ha/year)
Tigray	Inda Silase–Axum	27,512	7165	6.55%	11.4
	Mekelle–Adigrat	8737	1700	1.55%	9.7
	Maychew	5018	1084	0.99%	13.3
	Humera	6162	1265	1.16%	8.8
	Sub-total		**11,214**	**10.25%**	**11.1**
Amhara	Gondar–Debre Tabor	26,331	8577	7.84%	14.7
	Weldiya–Dese–Kemise	22,262	5576	5.10%	15.0
	Bahir Dar–Debre Markos	38,317	13,804	12.62%	17.7
	Sub-total		**27,958**	**25.55%**	**16.1**
Amhara & Oromia	North Shewa	27,180	9061	8.28%	15.0
	Sub-total		**9061**	**8.28%**	**15.0**
Oromia	South & West Shewa–Shambu	39,186	14905	13.62%	21.0
	Jimma & Metu	24,407	7914	7.23%	29.7
	Adama–Arsi–Robé	33,145	10,967	10.02%	16.8
	Sub-total		**33,786**	**30.88%**	**21.9**
Oromia & Somali	East & West Hararghe	22,351	7896	7.22%	18.3
	Sub-total		**7896**	**7.22%**	**18.3**
Oromia & SNNP	Hawassa–Shashemene	22,198	5963	5.45%	25.0
	Sub-total		**5963**	**5.45%**	**25.0**
SNNP	Gurage–Hosaina–Wolayita	19,864	9975	9.12%	22.7
	Sub-total		**13,542**	**12.38%**	**22.7**
Grand Total			**109,421**	**100.00%**	**19.4**

Source: Copernicus Global Land Service (CGLS 2019)

Table 2 shows that biomass production per ha is the highest in the clusters Jimma–Metu, Hawassa–Shashemene, Gurage–Hosaena–Wolayita, and South and West Shewa–Shambu. The main reasons for this high production per ha are rainfall and length of growing season. The clusters with largest total biomass production, due to their large surface areas, are South and West Shewa–Shambu, Bahir Dar–Debre Markos, and Adama–Arsi–Robé (Table 2).

Figure 7 shows that cropping is the main land use type in most of the selected clusters. This means that in most clusters, by-products of arable crops will be the main fodder ingredient for dairy cows. In many clusters, this will be straw from teff or other cereals. Only four clusters have a fair amount of grassland (herbaceous vegetation). Figure 8 combines data on three characteristics for potential development of the dairy sector. These are as follows:

- The THI, an indicator for heat stress in cows which is strongly related to main category b (environmental conditions for cows) in Table 1.
- Dry matter productivity (DMP), an indicator for the biomass production per ha. This indicator is strongly related to main category a (feed availability) in Table 1.
- Percentage of agricultural land cover. This is an indicator for the percentage of land used for agriculture. Low percentages denote limited agricultural activities, limiting the availability of local crop residues and by-products for dairy production.

The combination of these three indicators delineates areas where dairy cattle can endure in terms of climatic conditions, and at the same time, their owners could easily access feed. Such areas are represented by the greenish shade in Fig. 8.

**Fig. 5:**
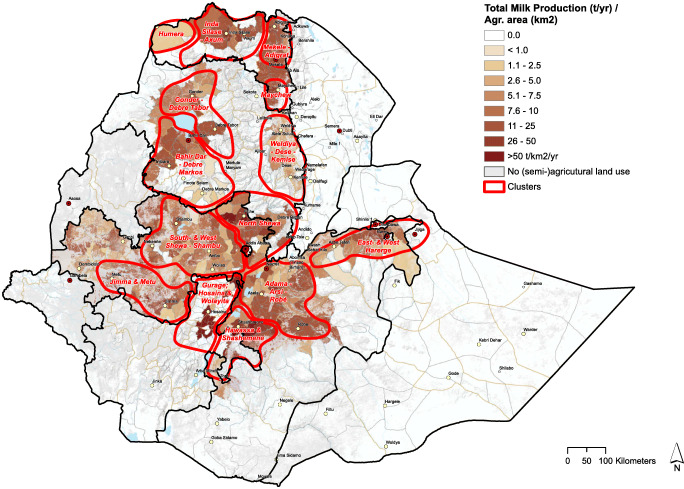
Total milk production per district within the clusters (2017 data)

**Fig. 6:**
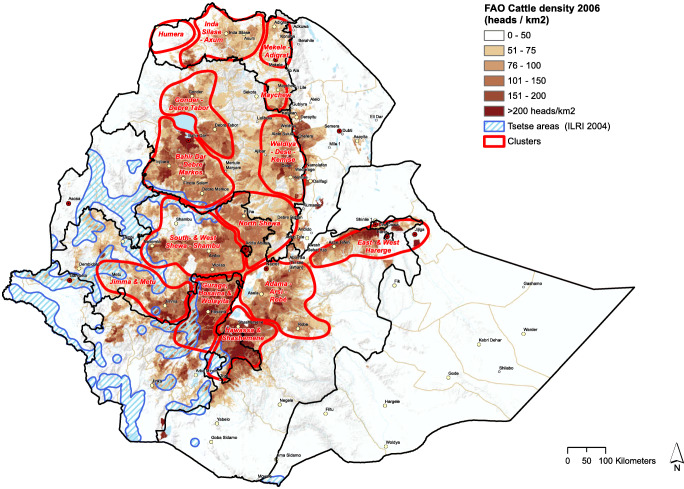
Cattle density and prevalence of tsetse (2006 and 2004 data FAO)

**Fig. 7:**
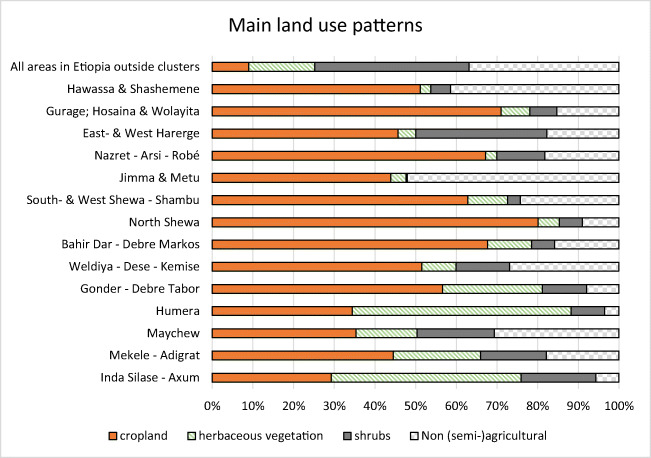
Land use patterns in the clusters

**Fig. 8:**
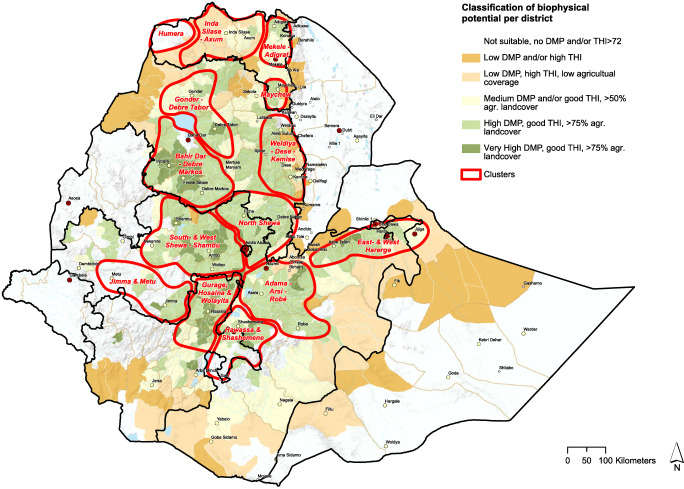
Combination of biophysical characteristics to delineate dairy production potential areas

From Figs. 4, 5, and 6, we see that cattle density per km^2^ in most cases is higher inside the selected clusters than outside them. Figures 4, 5, 6, and 8 clearly carve out the central part of the country as being a very high potential area for milk production. This covers a wide belt from the north, between Gondar and Mekelle, running southwards to Hawassa and Robé. In this belt, suitable temperatures and humidity limit heat stress in cows, more arable land is available, and crop productivity is relatively high. This translates to higher fodder yields and/or increased availability of crop residues and by-products for feeding cows. Figure 7

**Fig. 9:**
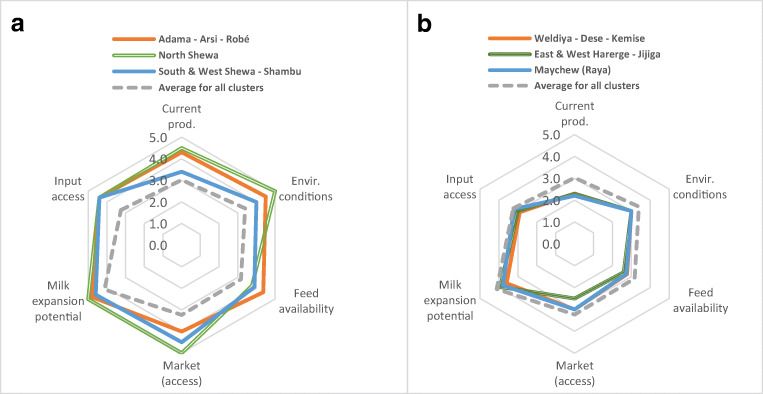
Comparison of clusters with average scores of all clusters. (**a**) Highest ranking clusters and (**b**) lowest ranking clusters

### Socioeconomic potential

This section assessed the dairy potential of all fourteen clusters based on two sources of data: (i) scoring of each cluster based on the 24 indicators of Table 2 and (ii) information from key informants on strengths and weaknesses of each cluster, as presented in Appendix 4. These results are futher elaborated in Figure 8 and in the next sections.

#### Cluster scores

Based on the average scores per category of indicators (Table 1), the North Shewa, Adama–Arsi–Robé, and South and West Shewa–Shambu clusters scored highest for potential development of their dairy sectors to increase milk production (Table 3). In Fig. 9, the results for these three clusters are benchmarked against the average of all fourteen clusters. All three clusters have a high rating for “feed availability” (category a in Table 3 and Appendix 3), which is the evaluation criterion with the highest weight. Meanwhile, these clusters also score very high on the indicators for expansion potential for milk volume, showing that there is still a strong possibility for growth in milk production in these clusters.

**Table 3:** Summarized scores on dairy cluster potential (see Appendix 3 for detailed overview) (score scale 0–5)

Region	Indicator category	Biophysical			Socioeconomic			Total overall score
		a	b	c	d	e	f	
		Feed availability	Environmental conditions for cows	Current production status	Access to inputs and services	Output market access	Milk production expansion potential	
	Score weighting (%)	35	10	20	10	10	15	**100**
	Cluster name	Cluster score (scale 0–5)						
Amhara & Oromia	North Shewa	3.8	5.0	4.5	4.4	5.0	5.0	**4.41**
Oromia	Adama–Arsi–Robé	4.4	4.5	4.3	4.4	4.0	4.8	**4.40**
Oromia	South & West Shewa–Shambu	3.9	4.0	3.4	4.4	4.5	4.6	**4.02**
SNNP & Oromia	Hawassa–Shashemene	3.1	3.5	3.4	3.5	4.0	4.5	**3.51**
Amhara	Bahir Dar–Debre Markos	3.5	3.0	3.1	3.5	3.5	4.5	**3.50**
SNNP	Gurage–Hosaena– Wolayita	3.2	3.5	3.0	3.0	3.5	4.5	**3.37**
Amhara	Gondar–Debre Tabor	3.0	3.5	3.6	3.1	3.5	3.9	**3.36**
Tigray	Inda Silase–Axum	3.2	4.0	2.7	2.9	2.5	4.5	**3.26**
Tigray	Mekelle–Adigrat	2.1	4.0	3.0	4.2	3.5	4.3	**3.14**
Tigray	Humera	4.1	2.0	3.3	1.5	1.5	3.8	**3.14**
Oromia	Jimma–Metu	3.5	3.5	2.6	2.7	2.0	3.6	**3.09**
Tigray	Maychew	2.7	3.0	2.2	3.2	3.0	3.8	**2.89**
Amhara	Weldiya–Dese–Kemise	2.8	3.0	2.3	2.9	3.0	3.6	**2.86**
Oromia & Somali	East & West Hararghe–Jijiga	2.6	3.0	2.3	3.0	2.5	3.9	**2.81**

The highest scoring clusters (Fig. 9a) are strongly focusing on the Addis Ababa market, which presently is the most viable and developed dairy market in the country. Meanwhile, the three lowest scoring clusters (Fig. 9b) in Oromia (stretching to Somali), Amhara, and Tigray show an attractive biophysical potential, though the current status shows a lower score for input and output market potential. A more detailed comparison of the strengths and weaknesses of various clusters is presented in Appendix 4.

## Discussions and recommendations

### Discussions

All identified clusters are within 100 km distance zones from cities with over 50,000 inhabitants, showing a linkage between potential production and possible markets for both inputs and outputs. Most of the clusters identified in this study are similar to those identified by Brandsma et al. (2013), confirming that the key milk production potential areas in Ethiopia have been in the neighborhood of Addis Ababa over the last years. This study added a few other clusters such as Humera and Inda Silase–Axum that were not characterized by Brandsma and others in 2013, while it splits some of the clusters into multiple clusters considering their source of input and output markets.

From Figs. 5 and 6, it is clear that the clusters closest to Addis Ababa have more dairy cows and currently produce more milk per square km (Table 1) than the clusters in other regions, which is associated to market proximity and the high urbanization level, although maps in Fig. 5 (total milk production per km^2^) and Fig. 6 (cattle density) show considerable overlap in areas within each cluster where cattle and milk are concentrated. This could indicate that the geographical distribution of dairy and beef cattle populations is closely related since areas with high beef cattle density also have a high dairy cattle density. Also since the data for both maps was collected nearly a decade apart from each other, the maps reveal that the development in cattle densities has been consistent over the past decade.

From Fig. 6, it is also clear that cattle density is very low in the areas where tsetse flies (category b, Table 1) are endemic, which reflects on cattle density and milk density. The tsetse predominance in the western part of Ethiopia was also found by other studies (Abera et al. 2018; Dagnachew et al. 2017). This explains the exclusion of some areas from the selected clusters: districts where the fly is present have few cows.

Table 3 shows that feed availability was low and could be a major issue in northern Ethiopia (except for Humera), conforming with findings of earlier studies (Gebrekidane et al. 2014; Berhane 2016; Alemneh 2019). The main sources for cattle feed in Ethiopia were identified by Bereda et al. (2017) and FAO (2018) to be mainly crop residues such as maize stover and straws of barley, sorghum, wheat, and teff. Despite the high feed availability in the Humera (Table 3), its biophysical potential is low in Fig. 8. This could be explained by a low share of agricultural area and the more severe heat conditions in this low-altitude area portrayed in Table 3 under environmental conditions for cows. Milk production in this area has been boosted by the large number of Begait cows, an indigenous breed that is highly adapted to the local weather conditions, consumes poor quality forage, but produces more milk per cow per year than other indigenous breeds (Mezgebe et al. 2017; Gebru et al. 2017).

Figure 8 illustrates the medium to low dry matter productivity and high THI conditions in the clusters Inda Silase–Axum and Mekelle–Adigrat, which shows that expansion of dairy farming in these areas would be challenging. Girma (2019) identified several inefficiencies in dairy production around these clusters and recommended that these could be overcome by improving farmers’ access to education and extension services as well as improving the enabling environment.

Milk production and processing is most strongly developed in areas in the proximity of Addis Ababa. Figure 8 shows that many other clusters have similar biophysical conditions as around Addis: high dry matter production, appropriate climate for higher producing dairy cows, and a large share of agricultural land delivering either grass or by-products from crops. Since dairy cows are already present in all clusters (Fig. 6), it is evident that other factors might hinder the development of milk production in some areas. For example, in addition to good climatic conditions for fodder production, fodder management skills are required by farmers to curb the challenge of dairy feed availability (Bekele et al. 2019).

In Amhara, the score for market is higher in Bahir Dar-Debre Markos as compared to Gondar-Debre Tabor (Table 3), as low reliability of markets in the latter is a major constraint to dairy production (Guadu and Abebaw 2016). This implies that there are opportunities for long-term development of dairy production and processing in such clusters. These clusters may have a relatively small local market as compared to their production potential and hence require better transport facilities with cooling options. Alternatively, milk produced from such areas could be processed into longer shelf life products and transported to other parts of the country.

### Methodological reflection

The approach of combining biophysical data and key informants assessments to identify and to estimate potential milk production areas was successful in generating large and useful datasets. The presentation in maps using several layers is an easy way to show different indicators in one picture, helping in demarcating high potential areas from low potential ones. Open GIS-databases can cost-effectively provide a large share of the required data. Additionally, the use of key informants creates opportunities for interaction between them, reducing bias and harmonizing outputs.

Despite the abovementioned advantages of the applied methodology, some weaknesses were also perceived:

- The collection of actual data on dairy cattle numbers and milk volumes within districts from the investigation area was very time-consuming. The data collected was incomplete, and in some cases, the data format varied between districts, making comparability difficult. This made us use more data from reputable open sources.
- The process to optimize and standardize the scoring of indicators in all the dairy clusters took more time than was expected. The lack of dairy specialists able to oversee many aspects of dairy production across all regions has complicated the process of standardizing the assessment scores. We concluded that, to make the process of scoring more efficient and to make the scoring more uniform, selected key informants should have a broad overview of the dairy sector, including knowledge of regional aspects of the clusters.
- The addition of strengths and weaknesses of the dairy clusters compensates for the somewhat limited expressiveness of the key informant assessment. We have therefore aligned the outcome of this assessment with the identified strengths and weaknesses.

Considering the aforementioned characteristics of this key informant approach, we recommend its application in other countries whenever a quick and cost-effective assessment of the potential of the dairy sector is desired. This should be adapted to the biophysical and socioeconomic context of the country and should also identify the strengths and weaknesses of each region.

### Recommendations

The developed assessment approach was successfully applied: the combination of data collection and key informant assessments leads to identification of clusters in Ethiopia with a potential to increase milk production in the future. These clusters have been ranked, and their strengths and weaknesses elaborated in such a way that both public and private investors could find the results useful in planning future investments in the dairy sector.

It will pave the way for future investments in other potential areas next to central part of the country where dairy production, processing, and marketing could be intensified. The study could also contribute knowledge that fosters public-private partnerships targeting the development of input and service provision systems, in order to fully realize the biophysical potential of identified dairy production clusters.

Companies seeking for opportunities in the long term may choose to invest in other clusters further away from Addis Ababa, which have a good milk production potential but less developed markets. This will require development of dairy infrastructure (a reliable milk transportation chain) and additional interventions to improve services for dairy farmers.

The applied methodology has strengths in its geographical presentation, using several layers of maps from open GIS databases to demarcate potential areas, and in its triangulation with key informant knowledge, which ranked and generated more information on strengths and weaknesses of specific clusters. This approach could also be adapted and applied in other countries or regions globally, whenever a cost-effective option for identifying areas for sustainable dairy or agricultural development is solicited.

## Supplementary Information

ESM 1 (DOCX 16 kb)

ESM 2 (PDF 402 kb)

ESM 3 (XLSX 24 kb)

ESM 4 (XLSX 25 kb)

## Data Availability

Additional data is provided in the Appendices.
