# Supplementary material for: Integrating knowledge on biophysical and socioeconomic potential to map clusters for future milk production in Ethiopia
Source: Trop Anim Health Prod. 2021 Apr 13;53(2):258. doi: 10.1007/s11250-021-02695-2 (PMC8043898; doi:10.1007/s11250-021-02695-2)
Supplement: Supplementary file 1 — (DOCX 16 kb) [file 11250_2021_2695_MOESM1_ESM.docx]

**Appendix 1. Description of the initial list of datasets and variables**

| **N.** | **Title** | **Resolution** | **List of variables** | **Link** |
| --- | --- | --- | --- | --- |
| **1** | World Climate data | 0.5° resolution | AIRTEMPERATURE ATMOSPHERICPHENOMENA ATMOSPHERICTEMPERATURE ATMOSPHERICWATERVAPOR CLOUDAMOUNT CLOUDCOVER CLOUDS FREQUENCY FROST MAXIMUM MINIMUMTEMPERATURE PRECIPITATION PRECIPITATIONAMOUNT VAPOURPRESSURE WATERVAPOR | <https://crudata.uea.ac.uk/cru/data/hrg/> |
| **2** | Soil grids | 250m | Absolute depth to bedrock (in cm) Depth to bedrock (R horizon) up to 200 cm Predicted probability of occurrence (0100%) of R horizon Bulk density (fine earth) in kg / cubic meter Cation exchange capacity of soil in cmolc/kg Clay content (02 micro meter) mass fraction in % Coarse fragments volumetric in % Texture class (USDA system) Soil organic carbon content (fine earth fraction) in g per kg Soil pH x 10 in H2O  Soil pH x 10 in KCl Sand content (502000 micro meter) mass fraction in % Silt content (250 micro meter) mass fraction in % Soil organic carbon stock in tonnes per ha Available soil water capacity (volumetric fraction)  Available soil water capacity (volumetric fraction) until wilting point Saturated water content (volumetric fraction) | <https://soilgrids.org/#!/?layer=ORCDRC_M_sl2_250m&vector=1> |
| **3** | ASTER GDEM/SRTM_dem | 90 m | elevation, slope | http://srtm.csi.cgiar.org/ http://asterweb.jpl.nasa.gov/GDEM.ASP |
| **4** | CGLS/AFRICOVER VITO | 100m | LCCS classification, with 23 main classes e.g. forests, grasslands, croplands, lakes, wetlands and 10 flexible fractional cover layers | <https://land.copernicus.eu/global/products/lc> |
| **5** | GRIDDED LIVESTOCK OF THE WORLD | 1km2 | distribution of bovine, small ruminants, pig and poultry species | https___www.livestock.geo_wiki.org_home_2__GRIDDED_LIVESTOCK_OF_THE_WORLD |
| **6** | DMP | 300m | DPM = overall growth rate or dry biomass increase of the vegetation, expressed in kilograms of dry matter/hectare/day (kg DM/ha/day) | https://land.copernicus.eu/global/products/lc |
| **7** | gROADSv1 |  | Road network and selected properties (name, type, width, etc.) | <http://sedac.ciesin.columbia.edu/data/set/groads-global-roads-open-access-v1> |
| **8** | OSM |  | Roadmaps, POI: in the study area, far better than Google Maps | <https://www.openstreetmap.org/search?query=Ethiopia> |
| **9** | GADM |  | GADM describes where these administrative areas are (the "spatial features"), and for each area it provides some attributes, such as the name and variant names. | <http://www.gadm.org/> |
| **10** | WDPA | km2 | (Distance to) all protected areas in the WDPA with IUCN classification. | [http://www.wdpa.org/ also: http://en.wikipedia.org/wiki/World_Database_on_Protected_Areas](http://www.wdpa.org/) |
| **11** | Global Human Settlement layer | 250m | Designation of urban centres, urban clusters and rural grid cells and population densities | Atlas of the Human Planet 2016: http://ghsl.jrc.ec.europa.eu/documents/Atlas_2016.pdf?t=1476360675 |
| **12** | gpwv4_2015 | km2 | Population density | <http://sedac.ciesin.columbia.edu/data/collection/gpw-v4> |
| **13** | jrc_acc_50k | km, travel time units | Travel time to major cities | <http://bioval.jrc.ec.europa.eu/products/gam/download.htm> |
| **14** | dist_allroads | Any resolution possible | Distance to roads | http://www.openstreetmap.org/; http://geoengine.nima.mil/ftpdir/archive/vpf_data/ |
