# Supplementary material for: Integrating knowledge on biophysical and socioeconomic potential to map clusters for future milk production in Ethiopia
Source: Trop Anim Health Prod. 2021 Apr 13;53(2):258. doi: 10.1007/s11250-021-02695-2 (PMC8043898; doi:10.1007/s11250-021-02695-2)
Supplement: Supplementary file 2 — (PDF 402 kb) [file 11250_2021_2695_MOESM2_ESM.pdf]

Appendix 2: Variables and maps for the delineation of dairy clusters

Biomass

Milk production with cows requires land for feed. This can be either grassland or cropland delivering fodder crops like maize and alfalfa or by-products from arable crops like teff, barley, oil seed plants, etc. A good proxy for fodder crop production is the estimate of biomass. Dry Matter Productivity (DMP), represents the overall growth rate or dry biomass increase of the vegetation, expressed in kilograms of dry matter per hectare per day (kg DM/ha). DMP is directly related to NPP (Net Primary Productivity). When combined with land cover classes for grassland and cropland producing fodder crops or by-products for animal feed, we can get to a reasonable estimate of the biomass available for animal feed. DMP can be estimated from satellites and other (meteorology) data. In our case, we used the product freely distributed in the context of the Copernicus Global Land Service, which has a 300 m resolution and a known (comparable with MODIS GPP) accuracy[1]. This was combined with the land cover described in the next section, selecting only (semi) agricultural areas. **Figure A2i** shows the cumulated annual values averaged for the years 2013-2014, with the milk cluster boundaries superimposed.

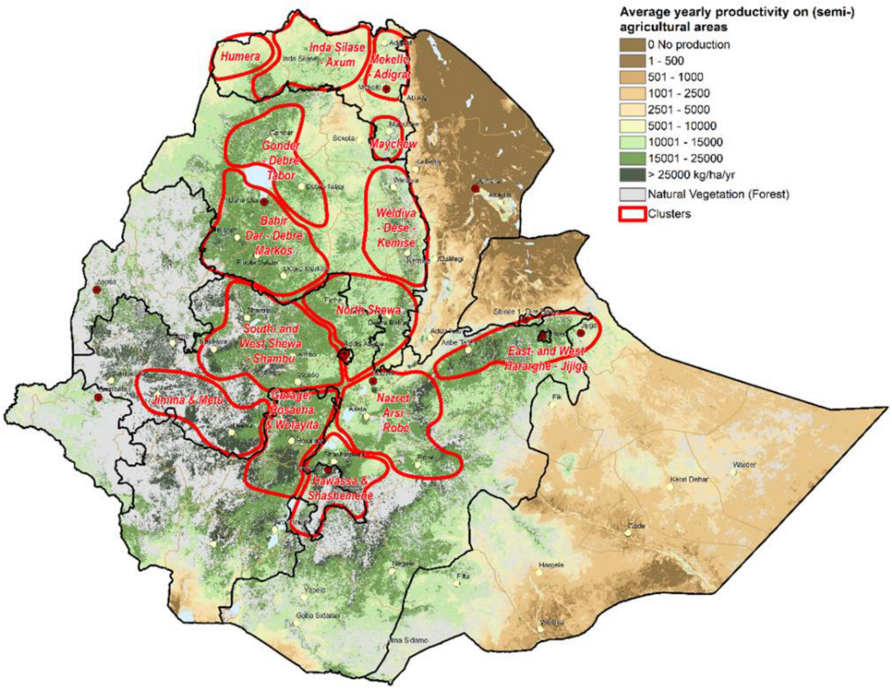

***Figure A2i**: Actual dry matter productivity (kg dry matter production per ha per year) from (semi-) agricultural areas*

Land cover

The land cover product we used is derived from data from another satellite (Proba-V) and distributed by Copernicus Global Land Service.[2] The land cover has a 100 m resolution and is based on the "Land Cover Classification System" (LCCS)[3] by FAO, with 23 main classes, e.g. forests, grasslands, croplands, lakes, wetlands and 10 flexible fractional cover layers (proportional estimates of vegetation cover for several land cover types). As mentioned in the previous section we are especially interested in the grassland and cropland land cover classes (or combinations of the above) which are the ones producing fodder crops or by-products for animal feed.

Heat stress vulnerability

During the summer months, heat stress affects dairy cows and other domestic animals in tropical, sub-tropical and sometimes temperate regions of the world. Heat and humidity create a sub-optimal condition for dairy cows to produce milk. The optimal thermal zone of dairy cows ranges from ca. 0°C to 22°C. If it becomes warmer, cows begin to alter their basal metabolism and metabolic rate. Combined with high humidity levels the effect of heat stress increases. The THI in **Figure A2ii** demonstrates how

[1] https://land.copernicus.eu/global/products/dmp

[2] https://land.copernicus.eu/global/products/lc

[3] http://www.fao.org/3/a-i5232e.pdf

the combined effects of temperature and relative humidity induce heat stress of dairy cows and the severity of heat stress. An index to estimate heat stress was developed combining relative humidity (vapour pressure in kPa) and monthly mean temperature (in °C). This index is called the Temperature Humidity Index (THI). Itis calculated as:

THI = (1.8xT + 32) – ((0.55 - 0.0055xRH) x (1.8xT - 26))

Where: T = Temperature in °C, and RH = Relative Humidity in %.[4]

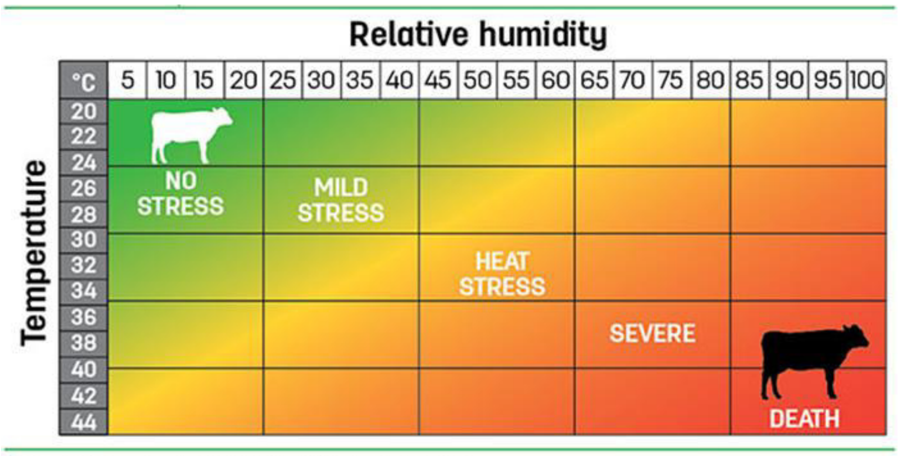

Livestock Conservation Institute (Whittier, 1993, Armstrong 1994)

Figure A2ii: Stress categories for dairy cows, based on temperature and relative humidity

Monthly mean temperature was combined with monthly vapour pressure data. Using these monthly values per km^2, the mean THI score per month was extracted.. High producing dairy cows begin to decline production at an average THI of 68. When the index ranges from 72 to 79, cows begin to suffer, and milk production drops rapidly. At THI of more than 80 cows become severely stressed and will not produce milk anymore. In many temperate regions of the world where summers are mild and temperature rarely exceeds 30°C, moderate to severe episodes of heat stress can occur due to high humidity. THI index of 75 or above can occur when temperature is 27°C, combined with a humidity above 80%. A second step was to aggregate these values per woreda[5] taking the monthly mean THI-values of all cells in the woreda. Based on the monthly THI scores per woreda, the maximum THI score per season was calculated. A final value per year is calculated as the average value over all four seasons. If the average maximum THI in four seasons >72 this is classified as "Very High THI scores", if THI is > 68 it is classified as "High THI scores"; all other classes are classified as "Good".

Classification of biophysical potential

The three factors above (biomass, heat stress vulnerability and land cover) were merged to generate a classification of the overall biophysical potential in terms of development of the dairy sector (**Figure 8**). The factors can be seen as proxy indicators for 1) environmental conditions for cows, 2) feed availability and 3) level of facilities to enable agriculture, hence the availability of local crop residues and by-products for dairy production.
Classes are: not suitable (no DMP and THI > 72); low DMP and High THI; low DMP, high THI and low agricultural coverage; low DMP, high THI and agricultural coverage > 50%; High DMP, good THI and >50% agricultural coverage; High DMP, good THI and >75% agricultural coverage; very High DMP, good THI and >75% agricultural coverage; and finally non-agricultural land use.

Additional verification variables

Additional supporting variables were also retained as possible means of verification of the dairy potential, namely distance to cities and cattle density. In **Figure 6,** we have mapped the Food and Agriculture Organisation (FAO) Cattle density 2006. Although these figures refer to total numbers of cattle and not to dairy cows, they were found to be in agreement with the biophysical potential classes.

[4] Climate data are derived from www.worldclim.org
[5] Woreda are the third-level administrative divisions of Ethiopia, equivalent to districts.
